# Dementia Care Across Long-Term Settings: Similar but Not Equal

**DOI:** 10.1093/geroni/igaa057.2318

**Published:** 2020-12-16

**Authors:** Sheryl Zimmerman, Christine Kistler, Jessica Scott, Kimberly Ward, Robin Zeigler, Louise Sullivan, Sarah Tomlinson

**Affiliations:** 1 University of North Carolina at Chapel Hill, Chapel Hill, North Carolina, United States; 2 North Carolina Department of Public Health and Human Services, Raleigh, North Carolina, United States; 3 Salve Regina University, Newport, North Carolina, United States

## Abstract

Nursing homes and assisted living (AL) communities are similar but not equal, and addressing the needs of residents with dementia differs across settings. It is important to appreciate that both settings are complex adaptive systems; as such, care intended to have widespread impact must be mindful of stakeholders, understand existing practices, and be pragmatic. This session will present an evidence-based program developed in nursing homes – Mouth Care Without a Battle, which teaches staff to provide daily, personalized mouth care to persons with cognitive and physical impairment – and considerations relevant for implementation in AL. Using data from more than 2,000 AL trainees and also AL administrators, supervisors, residents, family members, and trainers, it will situate findings in the context of implementation science and the NIH Stage Model, thereby making them applicable to any dementia care practice regardless its focus and the setting in which it is to be used.

